# A green approach to dual-drug nanoformulations with targeting and synergistic effects for cancer therapy

**DOI:** 10.1080/10717544.2016.1228716

**Published:** 2017-02-03

**Authors:** Shichao Wu, Xiangrui Yang, Yue Lu, Zhongxiong Fan, Yang Li, Yuan Jiang, Zhenqing Hou

**Affiliations:** 1 Research Institute for Biomimetics and Soft Matter, College of Materials, Xiamen University, Xiamen, China and; 2 The Department of Physics, Changji University, Changji, China

**Keywords:** Dual-drug, green, targeting, anticancer, nanoformulation

## Abstract

Exploration of efficient dual-drug nanohybrids, particularly those with high drug loading, specific targeting property, and long-termed stability, is of highly importance in cancer therapy. A pH-driven coprecipitation was performed in the aqueous phase to obtain a dual-drug nanoformulation, composed of 10-hydroxycamptothecine (HCPT) nanoneedles integrated with an exterior thin layer of the methotrexate (MTX)–chitosan conjugate. The high stability of nanohybrids in water and the targeting property provided by the MTX ingredient function synergistically to the prolonged and sustained drug release property in tumor tissues and the increased cellular uptake. The cytotoxicity test illustrates that dual-drug nanoneedles possess the remarkable killing ability to HeLa cells with the combination index at 0.33 ± 0.07. After cellular internalization, the release of both drug ingredients results in an excellent anticancer activity *in vivo* with the minimized adverse side effects. Design of a green approach to the carrier-free, dual-drug nanoformulations enables to develop emerging drug delivery systems for cancer diagnosis and treatment.

## Introduction

Drug delivery systems aim at producing prolonged circulation and efficient accumulation in tumor cells. Meanwhile, they could result in the safe and enhanced inhibition of cancer growth and the minimized adverse side effects. Nevertheless, exploration of efficient formulations for cancer therapy remains challenging. Among various treatment methods, codelivery of multiple functional agents is particularly promising to address the issue of drug resistance in cancer cells (Gottesman, 2002; Noguchi et al., 2009), and hence, to improve anticancer efficacy accordingly (DeVita et al., 1975; Al-Lazikani et al., 2012; Bahadur & Xu, 2012). It is known that each anticancer agent in the codelivery system can have the specific activity on cancer cells at the different growth stage with proper inhibition mechanism (Chabner & Roberts, 2005; Jia et al., 2009; Lehar et al., 2009). Hence, the synergistic effect of the codelivery system can potentially lead to significant therapeutic efficiency (McDaid & Johnston, 1999; Calabro et al., 2009). Moreover, insertion of a tumor-targeting agent, such as folic acid (FA) (Anderson et al., 1992; Weitman et al., 1992), transferrin (Choi et al., 2010) and other monoclonal antibodies, (Kocbek et al., 2007) in the codelivery system can enhance the therapeutic efficiency further. Interestingly, we note that some anticancer drugs like methotrexate (MTX) can function as a tumor-targeting agent as well (Duthie, 2001). Promisingly, interfacial integration of MTX with another anticancer drug in the nanohybrid could result in the dual-drug delivery system with improved therapeutic efficiency, compared with that of traditional formulations (Rosenholm et al., 2010; Jia et al., 2014).

Dual-drug delivery systems are usually fabricated as nanoformulations to overcome toxicity and the poorly-controlled dosing of traditional systemic combination therapies (Patil et al., 2010; Ma et al., 2013; Yan et al., 2013; Liao et al., 2014). Among various nanoformulations, nanocarrier-based dual-drugs have been the focus of related researches in the past decade, mainly due to the fast development of synthetic approaches to nanocarriers and corresponding interfacial modification tools for enhancing the stability (Martello et al., 2000; Ahmed et al., 2006; LoRusso et al., 2012). To date, numerous nanoparticulate carriers based on liposomes (Batist et al., 2009; Ashley et al., 2011; Park et al., 2012), vesicles (Holme et al., 2012; Song et al., 2012), polymers (Sengupta et al., 2005; Wang et al., 2006; Aryal et al., 2010; Kolishetti et al., 2010; Wang & Ho, 2010), polymer–drug conjugates (Li & Wallace, 2008; Lammers et al., 2009; Wang et al., 2013), mesoporous hybrids (Chen et al., 2009; Meng et al., 2010), iron oxide nanoparticles (Dilnawaz et al., 2010), etc. have been explored to deliver therapeutic drugs with excellent efficacy. Such nanocarriers can improve the pharmacokinetic effect significantly with the enhanced stability of anticancer drugs and the prolonged circulating half-period. Nevertheless, the main drawback of this approach lies in low drug loading because only a limited amount of drugs can be involved to a nanocarrier (Sengupta et al., 2005; Wang et al., 2006; Li & Wallace, 2008; Lammers et al., 2009; Aryal et al., 2010; Kolishetti et al., 2010; Wang & Ho, 2010; Wang et al., 2013). Exploration of carrier-free dual-drug nanoformulations which can provide a valuable approach to those with high drug loading remains technically challenging (Zhou et al., 2013). For instance, numerous nanoformulations containing amorphous dual-drug nanoparticles exhibit excellent cancer therapy efficacy (Huang et al., 2014; Chen et al., 2015; Zhao et al., 2015). Nevertheless, the metastable amorphous form could hamper long-termed stability of dual-drugs. Alternatively, conjugation of hydrophilic and hydrophobic drugs led to spontaneous formation of dual-drug micelles in water, relying on the pair of specific chemical functional groups on each drug compound (Huang et al., 2014). In another case study, coprecipitation of three drugs led to crystalline hybrid nanorods (Barua & Mitragotri, 2013). However, absence of an exterior protecting layer may cause the gradual aggregation of nanoparticulate hybrids and the deceased retention time. Hence, development of an efficient approach to achieve dual-drug nanoformulations with high stability, the targeting property, and most importantly, the significant therapeutic effect remains challenging. Meanwhile, exploration of a green approach to fabrication of dual-drug nanoformulations can have a profound impact on sustainable drug manufacturing.

In the current study, an aqueous dual-drug nanoformulation, composed of 10-hydroxycamptothecine (HCPT) and the MTX–chitosan conjugate, was fabricated in a green coprecipitation process driven by the abrupt pH switch in the aqueous mixture. The hybrid nanoneedles are characteristic of bearing a nanocrystalline HCPT core integrated with a MTX–chitosan conjugated shell, the latter of which also functions as a targeting agent and stabilizer of the dual-drug nanoneedles in water. Nanohybrids with high HCPT loading showed the prolonged and sustained release property due to the presence of the conjugated protection layer. In cytotoxicity tests, the nanohybrids exhibited an excellent killing ability to HeLa cells, which evidenced a synergistic effect of both drug ingredients and the targeting property of the MTX ingredient in the exterior conjugated layer. After cellular internalization, the release of active ingredients from dual-drug nanohybrids led to an excellent anticancer activity *in vivo*, and meanwhile, the minimized adverse side effects. Our novel approach highlights great prospect of green (nano)crystal engineering approaches for fabrication of carrier-free dual-drug nanoformulations with high therapeutic efficacy, which can find numerous applications in cancer diagnosis and treatment.

## Methods

### Materials

All chemicals were analytical grade and used as received without further puriﬁcation. 10-Hydroxycamptothecine (purity > 99%) was purchased from Lishizhen Pharmaceutical Co., Ltd. (Hubei, China). Methotrexate and folate (FA) were purchased from Bio Basic Inc. (Markham, ON, Canada) 1-(3-Dimethylaminopropyl)-3-ethylcarbodiimide hydrochloride (EDC) and N-hydroxysuccinimide (NHS) were purchased from Sigma-Aldrich. Chitosan (Mw = 70 000, 90% degree of deacetylation) was obtained from Zhejiang Aoxing. The BALB/C nude mice were purchased from Wushi Animal Trade Co., Ltd. (Jiangsu, China). Deionized (DI) water was used in all experiments.

### Synthesis of the MTX–chitosan conjugate

Methotrexate (10 mg) and chitosan (20 mg) were added in a volume of 2 mL PBS buffer solution (pH = 7.4) and stirred at rt for 10 h to obtain the MTX–CHITOSAN suspension. Afterwards, the suspension was dialyzed against a buffer solution (pH = 10) to remove free MTX molecules. The remaining suspension was centrifuged at 5000 rpm and lyophilized for 24 h to obtain the dry powder.

### Preparation of the dual-drug dispersion and HCPT–chitosan one

Ten micrograms of HCPT powder was dissolved in 200 μL NaOH aqueous solution (0.1 M, solution A), and 10 μg MTX–chitosan powder was dissolved in 200 μL HCl (0.1 M) to obtain the solution B. Afterwards, the solution A was added dropwise into the solution B under vigorous stirring for 1 min, and the mixture was sonicated (power 200 W) in an ice bath for 5 min. The suspension was centrifuged at 10 000 rpm for 5 min to remove impurities and lyophilized for 24 h to obtain the dry powder. For preparation of HCPT–chitosan dispersion, the chitosan solution was used to replace the solution B.

### Characterization

Morphology of the nanoneedles was examined by SEM (UV-70) at 10 kV. The zeta-potential values were determined with a Malvern Zetasizer Nano-ZS machine (Malvern Instruments, Malvern) at 25 °C under suitable dilution conditions. The average values were determined by three parallel measurements. Crystallinity of dual-drug nanoneedles was analyzed with XRD (X’pert PRO, Bruker D8 Advance, Germany). The X-ray diffractogram was scanned with Cu-ka radiation generated at 30 mA and 40 kV. The diffraction angle was from 5 to 60° with a step size of 0.016°.

The content of MTX in the dual-drug nanoneedles was determined by UV spectrophotometry (Beckman DU800). All samples were assayed at 305 nm. The calibration curve was drawn beforehand for determining the MTX concentration.

The content of HCPT in the dual-drug nanoneedles was determined by fluorescence spectrophotometry. All samples were assayed at 383 nm. The calibration curve was drawn beforehand for determining the concentration of HCPT. The content and entrapment efficiency are calculated by Equations (1)–(4):

(1) Drug loading content of HCPT (%)   =(weight of HCPT in nanoneedles)/      (weight of nanoneedles)×100% 

(2) Entrapment efficiency of HCPT(%)   =(weight of drug in nanoneedles)/      (weight of feeding drug)×100%

(3) Percentage of MTX in the conjugation (%)   =(weight of MTX in conjugation)/      (weight of conjugation)×100% 

(4) Drug loading content of MTX (%)   =(1-drug loading content of HCPT)      ×percentage of MTX in the conjugation×100%

### *In vitro* drug release studies

The *in vitro* drug release studies of nanoformulations were performed using the dialysis technique. The nanoneedles were dispersed in a PBS buffer solution (10 mL) and placed in a pre-swelled dialysis bag (MWCO 3500 Da). The dialysis bag was then immersed in 0.1 M PBS (200 mL; pH 7.4) and oscillated continuously in a shaker incubator (100 rpm) at 37 °C. All samples were assayed by a HPLC method. The released MTX was assayed using a HPLC (Waters Associates, Milford, MA) system consisting of a Waters 2695 Separation Module and a Waters 2996 Photodiode Array Detector (Hypersil ODS column 250 mm × 4.6 mm, 5 μm; 25 °C; elution flow rate at 1.0 mL/min; detection wavelength at 303 nm; HPLC grade acetonitrile and 40 mM potassium dihydrogen phosphate (pH 4.5) (volume ratio of 12/88) as the mobile phase.

### Confocal imaging of cells

Methotrexate was labeled with fluorescein isothiocyanate (FITC) molecules via a thiourea linkage to function as a fluorescent probe. Confocal imaging of cells was performed using a Leica laser scanning confocal microscope. Imaging of HCPT was carried out under the 382 nm laser excitation, and the emission was collected in the range of 500–550 nm. Imaging of FITC was carried out under the 488 nm laser excitation, and the emission was collected in the range of 500–550 nm. HeLa cells were seeded and preincubated at 37 °C for 24 h (5% CO_2_) before incubated with the FITC-labeled nanoneedles for 8 h. In contrast experiments, HeLa and MG-63 cells were incubated with nanoneedles or other chemicals under the same condition before confocal imaging. All cells were washed twice with a PBS buffer before imaging.

### Cellular uptake measured by fluorescence measurement

HeLa cells were seeded in a 24-well plate (5 × 10^6^ mL/well). The plate was then incubated at 37 °C for 24 h in a humidified atmosphere (5% CO_2_). The cells were then incubated with equivalent concentrations of nanoneedles (three recipes were dual-drug ones, HCPT–chitosan, and dual-drug nanoneedle in the presence of FA). The drug-treated cells were incubated for pre-determined time at 37 °C, followed by being washed twice with cold PBS, and digested by the trypsin (0.05%)/EDTA treatment. The suspensions were centrifuged at 1000 rpm at 4 °C for 4 min. The supernatant was discarded and cell pellets were washed with PBS to remove the background fluorescence in the medium. After two cycles of washing and centrifugation, cells were resuspended with 2 mL PBS and disrupted by vigorous sonication. The amount of HCPT in the sonicated mixture was analyzed using fluorescence spectroscopy (excitation at 382 nm). Blank cells sample in the absence of drug nanocrystals was measured to determine the cells auto-fluorescence level as the control.

### Cytotoxicity assays

The cytotoxicity of various particles mentioned earlier in this manuscript was determined by the MTT assay. Briefly, an adequate number of exponential phase HeLa cells was plated in quintuplicate in a 96-well flat bottomed microplate and incubated for 24 h in the culture solution in the presence of drug particles. In this study, a volume of 20 mL 3-(4,5-dimethyl-2-thiazolyl)-2,5-diphenyl-2-H-tetrazolium bromide (MTT) solution (5 mg/mL in PBS) was added in each well, and the plates were incubated at 37 °C for another 4 h. Afterwards, a volume of 150 mL dimethylsulfoxide (DMSO) was added, and the plate was agitated on a water bath chader at 37 °C for another 30 min. The absorbance at 570 nm was measured using a Microplate Reader (model 680; Bio-Rad).

To determine inhibitory drug concentrations (IC50) to stop 50% cell growth, dose response curves of HCPT, MTX, the HCPT–MTX mixture and the dual-drug nanoformations were performed. From the resulting curves of individual drug treatment and the dual-drug nanoformations effects, the combination index (CI) for the dual-drug nanoformations was calculated using the Chou–Talalay method:^50, 51^

$$\mathrm{CI}\;=\frac{{\mathrm{IC}}_{50}\;\mathrm{of}\;\mathrm{HCPT}\;\mathrm{in}\;\mathrm{the}\;\mathrm{dual}\;\mathrm{drug}\;\mathrm{nanoformations}}{{\mathrm{IC}}_{50}\;\mathrm{of}\;\mathrm{HCPT}}\;+\frac{{\mathrm{IC}}_{50}\;\mathrm{of}\;\mathrm{MTX}\;\mathrm{in}\;\mathrm{the}\;\mathrm{dual}\;\mathrm{drug}\;\mathrm{nanoformations}}{{\mathrm{IC}}_{50}\;\mathrm{of}\;\mathrm{MTX}}$$

In this analysis, synergy is defined when CI < 1 and the smaller the CI is, the stronger the synergy is.

### Biodistribution

For *in vivo* fluorescence imaging, DiR, a near-infrared fluorescent probe, was encapsulated into the free HCPT, HCPT–chitosan nanoneedles, and dual-drug nanoneedles. DiR–HCPT, DiR–HCPT–chitosan nanoneedles, and DiR-dual-drug nanoneedles were intravenously administered into the HeLa tumor-bearing nude mice via the tail veins at an equivalent dose of 1.0 mg DiR–HCPT per kg mouse body weight. At predetermined time intervals, the mice were anesthetized with 2.5% isoflurane and imaged using the Maestro *in vivo* imaging system (Cambridge Research & Instrumentation, Woburn, MA). After 24 h, the mice were sacrificed, and the tumor and major organs (spleen, liver, kidney, lung and heart) were excised, followed by washing the surface with 0.9% NaCl for the *ex vivo* imaging of DiR fluorescence using a Maestro *in vivo* imaging system.

### Tumor inhibition *in vivo*

When the HeLa tumor volume was approximately 60 mm^3^, the mice were randomly divided, and treated by intravenous injection of 0.9% NaCl, the crystalline HCPT and MTX mixture, the mixture of HCPT–chitosan nanoneedles and crystalline MTX, and dual-drug nanoneedles every 3 d at a dose of 80 μg HCPT and 10.1 μg MTX per mouse. The tumor volume and body weight were monitored every 3 d. The tumor volume was calculated by the following formula: tumor volume = 0.5 × length × width^2^.

After 21 d, the mice were sacrificed and the tumors were excised and weighed. Next, the tumors were fixed in 4% paraformaldehyde overnight at 4 °C, embedded in paraffin, sectioned (4 μm), stained with hematoxylin and eosin (H&E) and examined using a digital microscopy system.

### Statistical analysis

The statistical significance of treatment outcomes was assessed using Student’s *t*-test (two-tailed); *p* < 0.05 was considered statistically significant in all analyses (95% confidence level).

## Results and discussions

The synergistic effect of the dual-drug nanoformulation relies largely on the form, content and position of each drug in the nanohybrid. The first drug candidate – MTX functions as an anticancer drug and targeting agent (Duthie, 2001). Its presence in the exterior layer of dual-drug nanohybrids can facilitate the cellular uptake by target cells theoretically. In the present study, the MTX–chitosan conjugate was employed to encapsulate the nanocrystalline core of HCPT in a dynamic coprecipitation process and meanwhile, functioned as the stabilizer in water and a tumor-targeting agent. The synthesis of the MTX–chitosan conjugate is as follows. First, an amidation reaction was performed to achieve the MTX–chitosan conjugate (Figure S1). The conjugation was confirmed by an FT-IR spectrum, where an apparent shoulder peak at 1562 cm^−1^ indicated the C=O stretching vibration of the amido group (Figure S2). The conjugation efficiency expressed as the molar ratio of MTX and the (de)acetylated unit of chitosan is as high as 28.6 ± 1.7% with quantitative UV–vis spectrophotometry. Notably, the value is conveniently tunable by employing different amidation periods. The conjugate, due to its molecular similarity to chitosan, shows good and poor solubility in acidic and neutral/basic aqueous phases, respectively.

**Figure 1.**
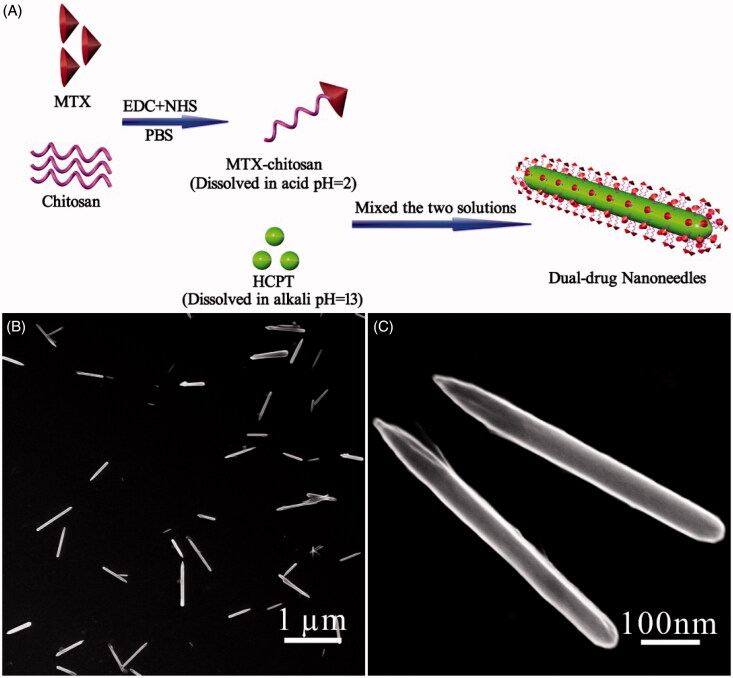
(A) Scheme illustrating the engineering approach to dual-drug nanoneedles. (B, C) SEM images showing overview (B) and structural details (C) of nanoneedles, EDC and NHS should be 1-(3-dimethylaminopropyl)-3-ethylcarbodiimide hydrochloride and N-hydroxysuccinimide, respectively.

The main drug in the dual-drug nanohybrid is HCPT – a hydrophobic anticancer drug soluble in numerous organic solvents (Hong et al., 2009; Wu et al., 2009). Its nanocrystalline form, protected by surfactants or polymers, was obtainable in an anti-solvent precipitation process with water as an anti-solvent in previous studies (Chen et al., 2015; Yang et al., 2015; Zhao et al., 2015). For instance, our recent study showed that a membrane emulsifier-assisted anti-solvent precipitation could be employed for integration of HCPT nanoneedles with a polymer layer to achieve comet-shaped microparticles with the sustained release property (Yang et al., 2015). We emphasize that HCPT has distinct formula at different pH values, *i.e.* the carboxylate and lactone forms under basic and neutral/acidic conditions, respectively (Tian et al., 2015). For instance, the gravimetric method confirmed that HCPT solubilities were 18.2 and 0.008 g/L when pH values were at 13.0 and 7.0, respectively. Hence, an abrupt pH change to 7.0 in a saturated HCPT solution (pH = 13.0) can immediately generate the supersaturation value over 2000, which is as high as that in a typical anti-solvent precipitation process.

(Nano)particulate-based dual-drug delivery systems require the prolonged and sustained release property, which can be achieved from surface modification. In a standard experimental set-up, the fast mixing of an alkaline HCPT solution (pH = 13) with an acidic MTX–chitosan one (pH = 2.0) in the presence of ultrasound led to coprecipitation of both ingredients. According to our recent study (Yang et al., 2015), we hypothesize that the growth of HCPT nanocrystals was halted as they were encapsulated by the coprecipitation of the MTX–chitosan conjugate (Figure 1A). The assumption is strongly supported by a zeta-potential value of +21.4 ± 2.1 mV, which demonstrates the interfacial presence of a positively-charged layer of MTX–chitosan conjugates on each nanoneedle. Hence, the exterior MTX–chitosan conjugate layer can function as the stabilizer of each nanoneedle in the aqueous phase. Interestingly, a 2 wt% dual-drug dispersion showed good stability for 2 d at least. The data in the current study clearly verifies that the stability of dual-drug nanohybrids can lead to the elongated retention time, facilitating the accumulation of anticancer drugs in tumor tissues and the subsequent cellular internalization. It is also interesting to know the HCPT form in the nanohybrid, which has a direct impact on drug delivery properties. The nanocrystalline form of HCPT was confirmed by the presence of sharp peaks in the XRD pattern (Figure S3). This can enhance their stability highly in comparison with its amorphous counterparts in previous nanoformulations (Tian et al., 2015). Meanwhile, amorphous nature of the MTX–chitosan conjugate was confirmed by the presence of two broad peaks in the same XRD pattern (Figure S3). Thus, the precipitation driven by an abrupt pH change in the aqueous phase is an emerging efficient and green alternative to anti-solvent ones for fabrication of dual-drug nanoformulations.

According to previous studies (Champion & Mitragotri, 2006; Champion et al., 2007; Gratton et al., 2008; Vacha et al., 2011; Barua et al., 2013), size and shape of nanodrugs can impact the extent and specificity of internalization. In a pH-driven coprecipitation procedure, numerous parameters need to be optimized to obtain the high-quality dual-drug nanoformulation. The optimized experimental conditions in the current study led to typical nanoneedles approximately 600 nm and 80 nm in length and width, respectively (Figure 1B,C). Notably, the ratio between HCPT and the MTX–chitosan conjugate is crucial for the shape control of nanoneedles. They aggregated to spherulitic microcrystals when the conjugate content was increased (Figure S4A,B). Conversely, limited conjugates were incapable of stabilizing HCPT nanocrystals from aggregation and precipitation thereafter (Figure S4C,D). A control experiment also showed that HCPT precipitation in the absence of the MTX–chitosan conjugate, nevertheless, resulted in rod-like bulk crystals over 10 μm in length (Figure S5).

Three additional parameters, namely the pH value, the HCPT concentration, and ultrasound, also played essential roles in determining the structure of the dual-drug. For instance, either decreasing the HCPT concentration or removal of ultrasound led to the increased size, which can be attributed to the decreased nucleation rate (Figure S4E). The increased particle size also accompanied slight aggregation assumedly due to the relatively slow coprecipitation kinetics. Furthermore, the final pH value in the mixture played an important role in the precipitation kinetics. Deviation of the pH value from seven deteriorated the dynamic encapsulation of growing HCPT nanocrystals with the MTX–chitosan conjugate, and hence, nanoneedle aggregates with the poor dispersibility were obtained (Figure S4F).

Additional characterization tools were applied to provide the compositional information of dual-drug nanoneedles. As the MTX–chitosan conjugate had no fluorescent signal, fluorescence spectroscopy could be used to measure the HCPT content in nanoneedles of 68.8 ± 2.5% (see measurement details in the SI material). As the remaining mass in nanoneedles is completely attributed to the MTX–chitosan conjugate, the calculation shows that the MTX loading is 8.9 ± 0.3%. Furthermore, the calculation, based on the quantitative fluorescence spectroscopy, indicated that the encapsulation efficiency of HCPT was about 80.1% in a coprecipitation process.

The *in vitro* drug release property of the dual-drug nanoformulation was evaluated with the dialysis technique. Either drug in the nanohybrid showed remarkable prolonged delivery properties compared with their individual counterparts (Figure S6). For instance, only about 10% MTX in dual-drug nanoneedles was released after 48 h; while the profile of MTX powders shows that over 30% of the drug was released within 1 h (Figure S6A). The prolonged release property of MTX in nanoneedles can be attributed to its conjugation with chitosan. Interestingly, the addition of protease effectively accelerated the releasing rate of MTX because it decomposed the conjugate and released free MTX into the dialysate (Figure S6A). Moreover, the MTX–chitosan conjugate also provided the protection for the encapsulated HCPT nanocrystals from fast dissolution (Figure S6B). The drug release curve also indicated that the dissolution of either drug was smooth, demonstrating the sustained release property of either drug. In short, the proper positioning of both ingredients, plus the conjugation of MTX with chitosan, caused the prolonged and sustained release properties of both ingredients in the dual-drug nanoformulation. The results are in line with our previous study, where the external polymer layer functioned similarly (Yang et al., 2015). We highlight prolonged and sustained drug release properties with the high value of drug loading in the current study.

**Figure 2.**
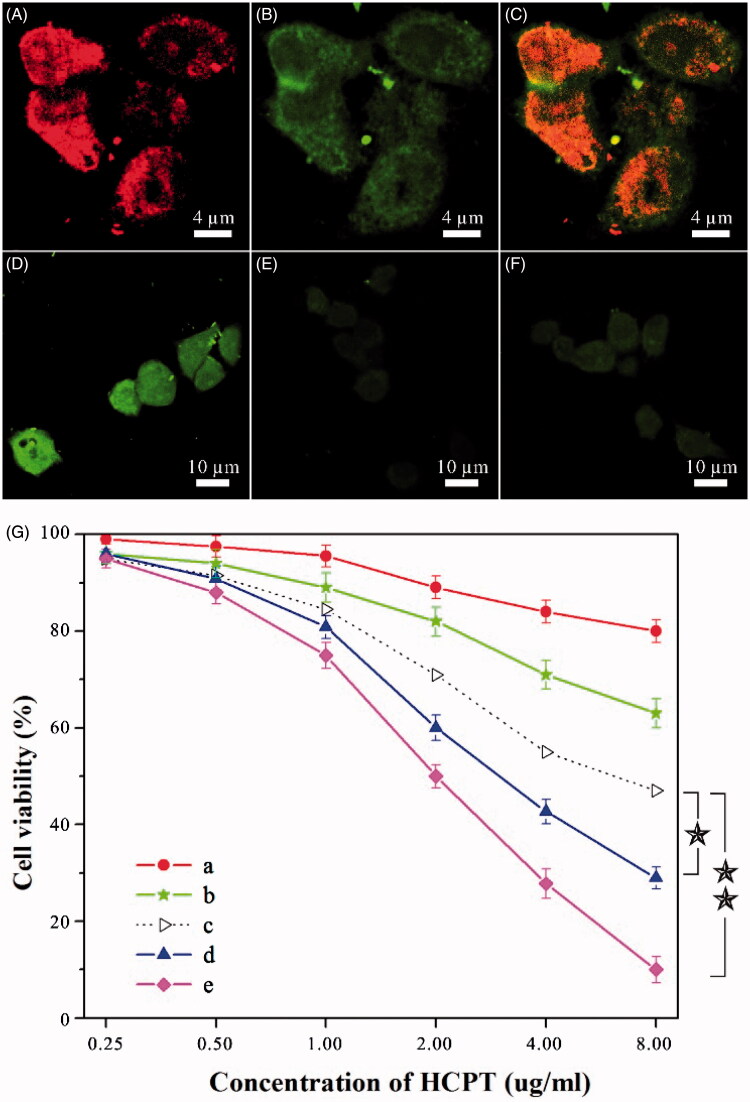
(A–F) Results of intracellular drug delivery in HeLa cells, which were incubated for 8 h at 37 °C. Images A–C, CLSM images of HeLa cells incubated with dual-drug nanoneedles. Nanoneedles were pretreated with FITC for imaging the MTX ingredient. The red (A) and green (B) fluorescent contrasts indicate the presence of MTX and HCPT, respectively. Image C is the combined image of images A and B. Images D–F, CLSM images of HeLa cells incubated with dual-drug nanoneedles (D), HCPT–chitosan nanoneedles (E) and dual-drug nanoneedles in the presence of FA (F). (G) *In vitro* cell viability of HeLa cells treated with the MTX–chitosan conjugates (a), HCPT–chitosan nanoneedles (b), the theoretical value of MTX–chitosan conjugates and HCPT–chitosan nanoneedles (c), the mixture of MTX–chitosan conjugates and HCPT–chitosan nanoneedles (d) and dual-drug nanoneedles (e) after incubation of 24 h. *p* < 0.05.

MTX, a tumor-targeting agent, can guide dual-drug nanoneedles for the cellular uptake by target cells. To access the cellular uptake, dual-drug nanoneedles were coupled with a fluorescent compound – fluorescein thiocyanate (FITC) beforehand for enhancing the visualization of MTX under confocal laser scanning microscopy (CLSM). After an incubation process, both red and green fluorescence signals could be visualized in HeLa cells. It was indicated that dual-drug nanoneedles could deliver and release both drugs into HeLa cells, allowing dual-drug nanoneedles for the synergistic anticancer treatment (Figure 2A–C). To evaluate the targeting property of MTX, HCPT–chitosan nanoneedles (HCPT = 64.7 wt%) were employed for a comparison test. The intense fluorescence emission of HCPT was detected in cells exposed to dual-drug nanoneedles, whereas weak signals were collected when HCPT–chitosan nanoneedles were used (Figure 2D–E; also see quantitative fluorescence data in Figure S7). This difference illustrates that integration of MTX in nanoneedles could effectively enhance the cellular uptake.

Next, functionality mechanism of MTX on cellular uptake is discussed. Two series of experiments were designed to ascertain whether the uptake endured a receptor-mediated endocytosis or bulk-phase one. First, the pretreatment of HeLa cells with excessive FA molecules suppressed the cellular uptake of the dual-drug nanoformulation effectively (Figure 2F). This suppression can be attributed to the affinity between FA and its corresponding receptors in target cells, which impedes internalization of dual-drug nanoneedles via the receptor-mediated endocytosis. Hence, weak signals attributed to the dual-drug should be generated via the bulk-phase endocytosis. We also note that the appearance of fluorescent signals in Figure 2(D) was apparently faster than those in Figure 2(F). This difference can be another evidence of bulk-phase endocytosis of nanoneedles in the presence of FA because the cellular internalization rate via the receptor-mediated endocytosis is faster than that of the bulk-phase one (Sahay et al., 2010). Another series of experiments were performed on the cellular uptake by MG-63 cells, which lacked FA receptors. Both dual-drug nanoneedles and HCPT–chitosan ones produced the similar cellular uptake, which hinted that both nanoneedles functioned via the bulk-phase endocytosis (Figure S8). In short, both groups of experiments unambiguously confirm that the targeting property of MTX is mainly due to its specific affinity to FA receptors.

The killing ability of dual-drug nanoneedles to cancer cells was studied thereafter. The cytotoxicity was evaluated using the methyl thiazolyl tetrazolium (MTT) assay with HeLa cells. Cytotoxicity of a physical mixture of HCPT–chitosan nanoneedles and the MTX–chitosan conjugate was higher than the theoretical value, which was calculated by counting the percentage of the cells killed by either ingredient (Figure 2G). This enhancement can be attributed to the synergistic effect of both ingredients, which was in accordance with previous studies (Soma et al., 2000; Patil et al., 2009; Markovsky et al., 2014). Moreover, the dual-drug nanoformulation significantly showed the higher cytotoxicity than that of the abovementioned physical mixture (Figure 2G). This further increase can be attributed to the targeting property of MTX, which is exclusively positioned on the exterior surface of dual-drug nanoneedles. Hence, the synergetic effect of both drugs and the targeting property of MTX facilitated the cytophagy and hence, caused the enhanced killing ability to cancer cells than those dual-drug delivery systems lacking targeting agents (Ahmed et al., 2006; Liao et al., 2014). To quantify the synergetic effect, the CI was calculated based on the Chou–Talalay equation (Chou & Talalay, 1984; Barua & Mitragotri, 2013). While the CI value of the physical mixture was 0.67 ± 0.06, that of the dual-drug nanoformulation was decreased to 0.33 ± 0.07, indicating the synergistic effect of dual-drug nanoneedles. Nevertheless, the presence of FA could undermine the killing ability of the dual-drug nanoformulation by deteriorating nanoneedle internalization via the receptor-mediated endocytosis (Figure S9).

**Figure 3.**
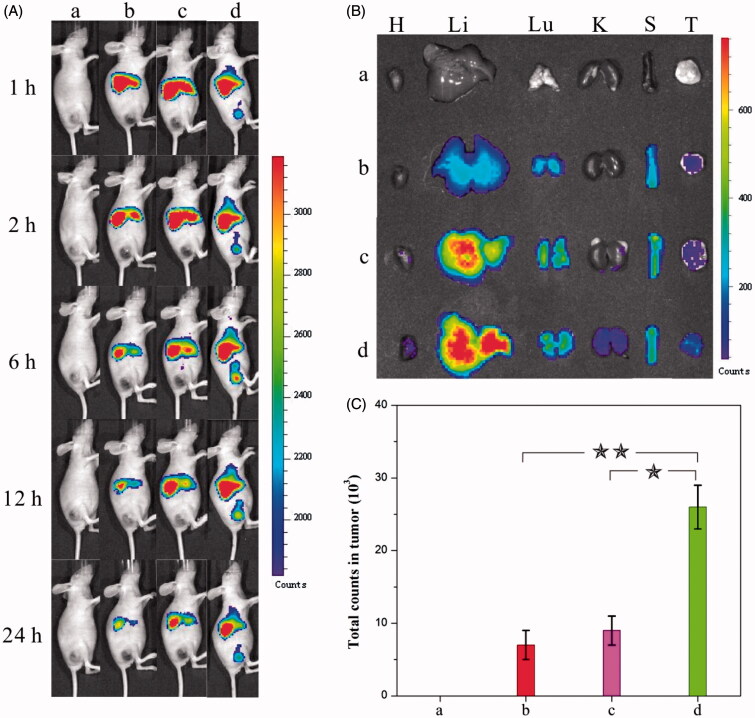
(A) Distribution and tumor accumulation of DiR-nanoparticles in HeLa tumor-bearing mice receiving intravenous injection of the indicated formulations. (B) *Ex vivo* fluorescence imaging of the tumor and normal tissues harvested from the euthanized HeLa tumor-bearing nude mice. The images were taken 24 h after the injection. H, Li, Lu, K, S and T represent heart, liver, lung, kidney, spleen and tumor, respectively. (C) DiR fluorescence intensity in tumor tissues collected at 24 h following systemic injection. *p* < 0.05. (a) 0.9% NaCl, (b) DiR-HCPT, (c) DiR-HCPT–chitosan nanoneedles and (d) DiR-dual-drug nanoneedles.

The (nano)formulations mentioned beforehand were subsequently employed in tumor treatments on mice to evaluate the efficacy. The *in vivo* biodistribution of HCPT, the HCPT–chitosan nanoformulation, and the dual-drug nanoformulation was studied beforehand to access their tumor-targeting ability, which was deemed a crucial factor to evaluate their anticancer capability. The treatment was performed by injecting a DiR-(nano)formulation intravenously into a mice bearing tumors derived from human cervical carcinoma HeLa cells (DiR was used as a near-infrared fluorescence probe). Afterwards, fluorescent images of mice were taken at different time intervals to compare the tumor-targeting effect of various (nano)formulations. Importantly, intense fluorescent signals were visualized at tumor areas in the dual-drug nanoformulation group compared with the relatively weak ones in comparison groups (Figure 3A). The intensity of the fluorescent signal in the tumor site increased gradually in the first 6 h, indicating the continuous and sustained accumulation of dual-drug nanohybrids in tumors. As comparison, the signals in the whole body of the same mouse decreased gradually in the first 24 h. After 24 h, the mice were sacrificed to collect total fluorescent counts in tumor and normal tissues from each group (Figure 3B,C). The fluorescence intensity in the tumor tissue treated with the dual-drug nanoformation was significantly higher than those in comparison groups. It was validated that introduction of MTX offered dual-drug nanoneedles excellent tumor-targeting efficacy. The targeting property of MTX – an anticancer drug itself in the current study exhibited the similar targeting effect to well-known ones used in previous studies (Anderson et al., 1992; Weitman et al., 1992; Das et al., 2006; Kocbek et al., 2007; Choi et al., 2010; Guo et al., 2013; Huang et al., 2014; Sun et al., 2015).

**Figure 4.**
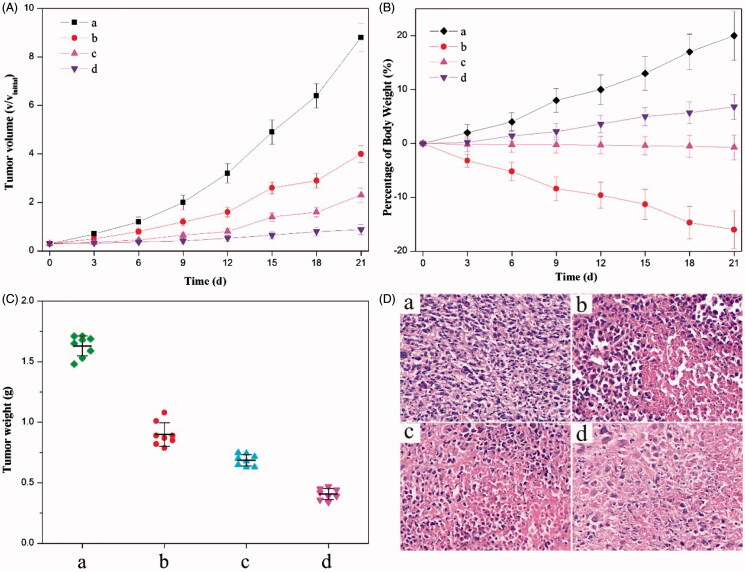
Anticancer effects of different (nano)formulations. (A) Volume change of tumor in mice during the treatment. (B) Weight change of the tumor-bearing mice during the treatment. (C) Weights of HeLa tumors after being treated by different (nano)formulations. (D) Histological section of the tumor of the mice after the treatment. (a) 0.9% NaCl aqueous solution, (b) the crystalline HCPT and MTX mixture, (c) the mixture of HCPT–chitosan nanoneedles and the crystalline MTX and (d) dual-drug nanoneedles. All HCPT–MTX formulations used the same concentration of HCPT and MTX in mice bearing HeLa tumor. *p* < 0.05.

Moreover, *in vivo* anticancer effects were investigated by evaluating the efficacy of tumor inhibition. Among all (nano)formulations tested, the dual-drug one provided the most pronounced inhibition effect on HeLa tumor xenografts generated in mice (Figure 4A). As comparison, other formulations composed of either the physical mixture of HCPT and MTX or that of HCPT–chitosan nanoneedles and MTX also showed the enhanced inhibition effect when compared with the control injection containing the 0.9% NaCl solution. Both the gravimetric method and the histologic image concluded that the tumor shrinkage was most impressive by using the dual-drug nanoformulation, which meant that it had the superior therapeutic efficacy to comparison groups (*p* < 0.05) (Figure 4C,D).

Application of an anticancer drug – HCPT was largely hampered by its high toxicity. For instance, listlessness/laziness and the severe body weight loss of mice occurred in the chemotherapy by using the physical mixture of HCPT and MTX for the anticancer treatment (Figure 4B). Regarding the treatment with a mixture of HCPT–chitosan nanoneedles and free MTX, there existed the small body weight loss of mice, illustrating that side effects were exceedingly mild, which mainly resulted from the small dose of free MTX. Delightedly, we note that employment of a dual-drug nanoformulation for tumor inhibition witnessed no obvious weight loss or other side effect. Hence, the dual-drug nanoformulation is a mild approach to the anticancer treatment with the superior efficacy to comparison (nano)formulations employed in the current study. Overall, the results clearly indicated that dual-drug nanoneedles with the significant anticancer effect and low toxicity would greatly improve the efficacy of cancer therapy.

## Conclusion

The current study presents a green approach, based on the pH-driven precipitation technique performed in the aqueous phase, to obtaining the HCPT- and MTX-based dual-drug nanoformulation for cancer therapy. The synergistic effect of both drugs in the nanoformulation provides a unique platform for designing dual-drug nanoformulations with high drug loading, the targeting property and imaging capability. The sustained and prolonged drug release property, plus the targeting property of the MTX ingredients, allows for effective cellular internalization and the enhanced cytotoxicity compared with either of the individual drug or their physical mixture. The current study highlights the feasibility of designing crystal engineering techniques for fabrication of dual or multiple drug nanoformulations with high stability compared with their amorphous counterparts. Particularly interestingly, the emerging precipitation method, based on the abrupt pH change instead of inclusion of any organic solvent, opens door for fabrication of nanoformulations in the aqueous phase to have a profound impact on sustainable drug manufacturing.

## Supplementary Material

Electronic_Supplementary_Information__ESI_.docx

## Notes

**Supplementary material available online**
