# Supplementary material for: A green approach to dual-drug nanoformulations with targeting and synergistic effects for cancer therapy
Source: Drug Deliv. 2017 Feb 3;24(1):51–60. doi: 10.1080/10717544.2016.1228716 (PMC8241172; doi:10.1080/10717544.2016.1228716)
Supplement: Electronic_Supplementary_Information__ESI_.docx [file IDRD_A_1228716_SM0219.docx]

**Electronic Supplementary Information (ESI)**

A Green Approach to Dual-Drug Nanoformulations with Targeting and Synergistic Effects for Cancer Therapy

*Shichao Wu,^a^ Xiangrui Yang,^a^ Yue Lu,^a^ Zhongxiong Fan,^b^ Yang Li,^a^ Yuan Jiang,^a^* and Zhenqing Hou^a,b^**

^a^Institute of Soft Matter and Biomimetics, College of Materials, Xiamen University, Xiamen 361005, China

^b^The Department of Physics, Changji University, Changji, 831100, China

E-mail: houzhenqing@xmu.edu.cn

**The file includes**

1. Figure S1-9
2. Table S1


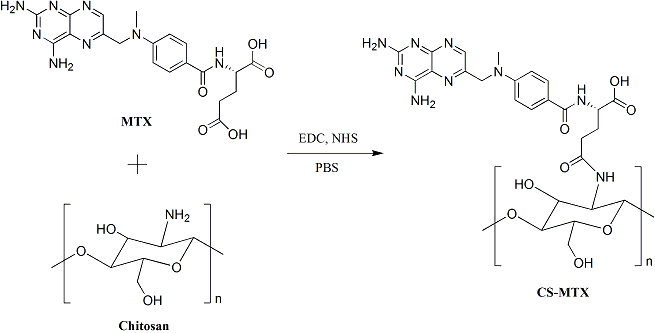


**Figure S1.** Synthetic route of the MTX-chitosan conjugate.


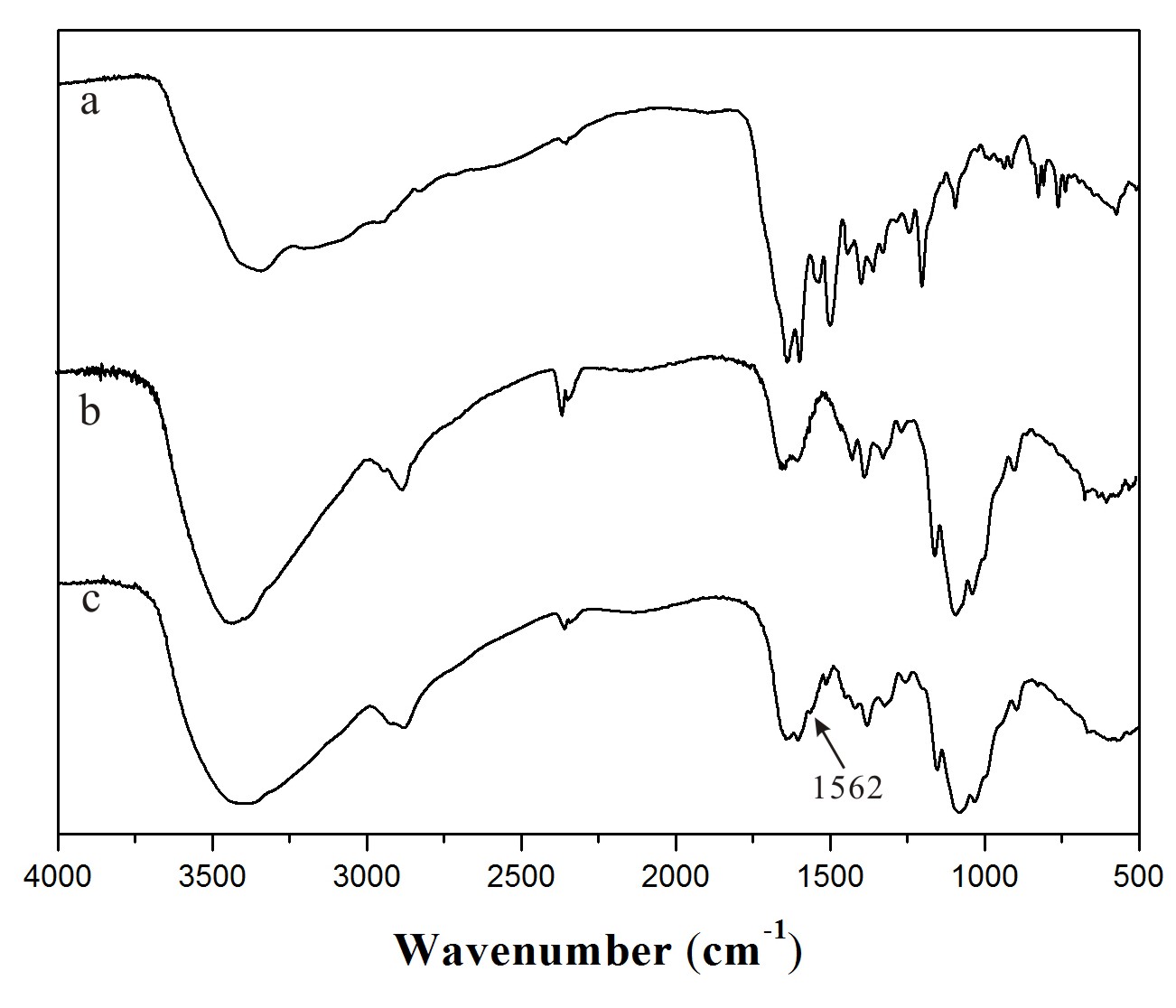


**Figure S2.** FT-IR spectra of MTX (a), chitosan (b), and the MTX-chitosan conjugate.


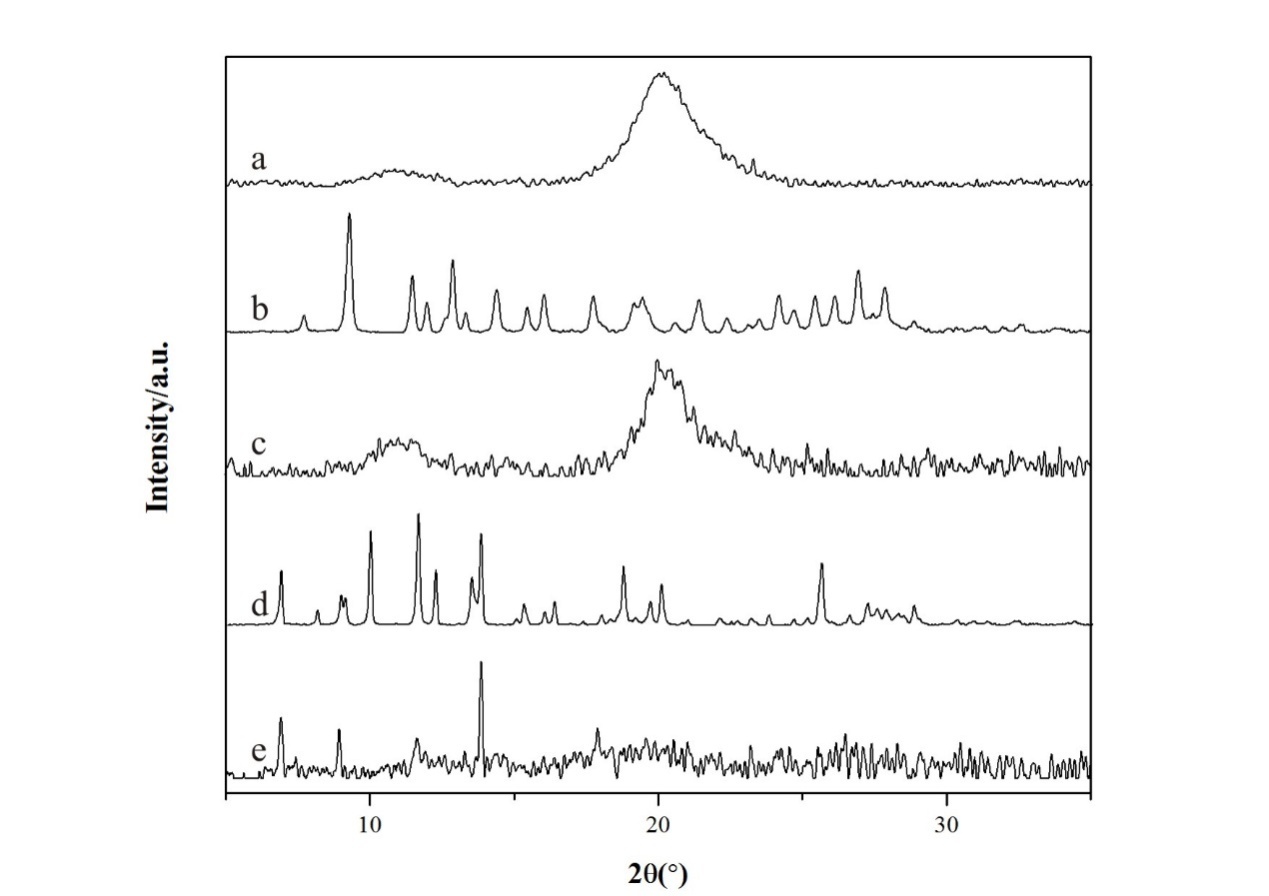


**Figure S3.** XRD patterns of chitosan (a), MTX (b), the MTX-chitosan conjugate (c), HCPT (d), and dual-drug nanoneedles (e).

**Table S1.** Experiment conditions in **Figure S4** in the manuscript.

| Groups | Factors | | | | |
| --- | --- | --- | --- | --- | --- |
|  | HCPT/conjugate  (*w/w*) | Ultrasonic power (W) | pH value | [HCPT] (µg/mL) | [conjugate] (µg/mL) |
| A–B | 10:1 | 200 | 7.0 | 50 | 500 |
| C–D | 1:10 | 200 | 7.0 | 500 | 50 |
| E | 1:1 | 0 | 7.0 | 50 | 50 |
| F | 1:1 | 200 | 6.0 | 50 | 50 |


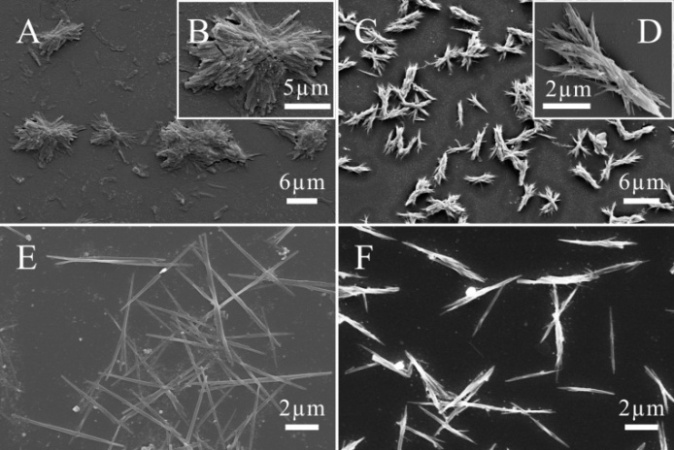


**Figure S4.** SEM images of dual-drug nanoneedles under different conditions. Images A-B and C-D show nanoneedle aggregates when the mass ratio of HCPT and the conjugate at 10:1 and 1:10, respectively. Image E shows nanoneedles were obtained when the initial HCPT concentration was low and no sonication was used. Image F shows nanoneedles were obtained when the fianl pH value was six. See experimental details of each image in Table S1.


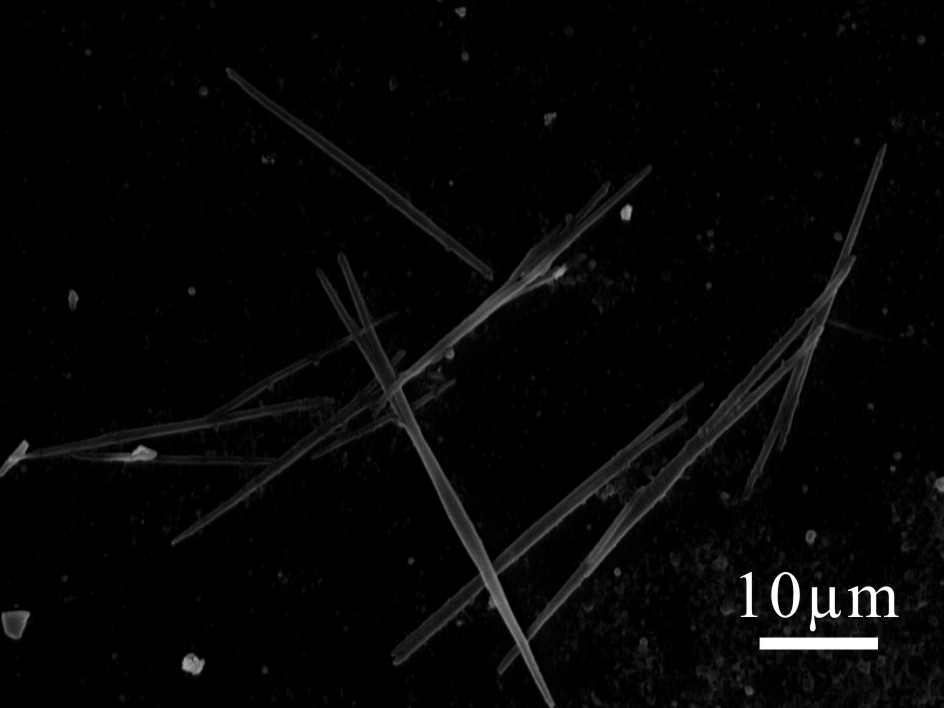


**Figure S5.** The SEM image of HCPT bulk crystals.


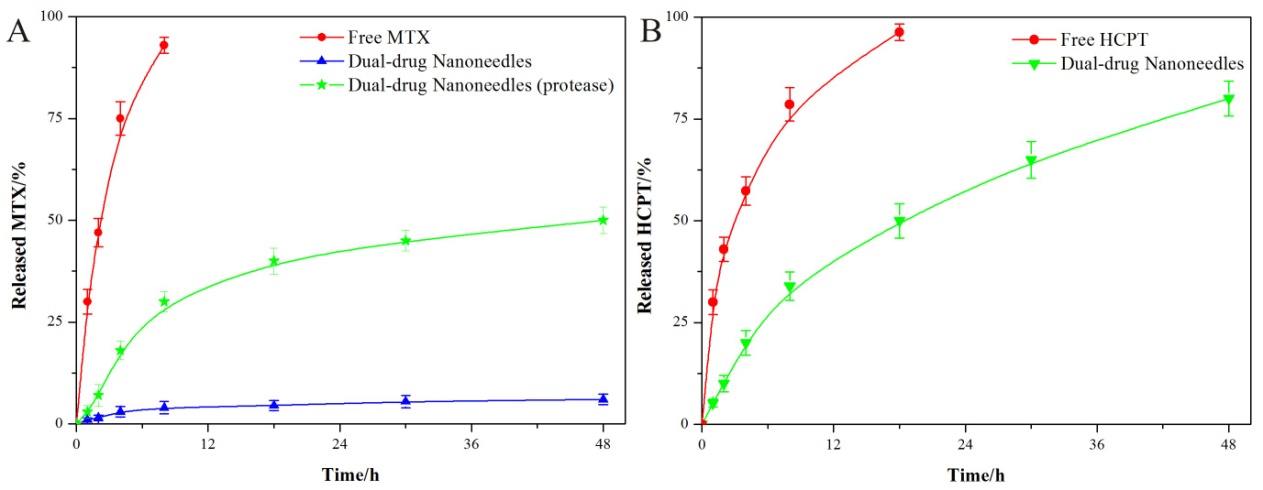


**Figure S6.** *In vitro* drug release profiles of dual-drug nanoneedles in PBS (pH 7.4) at 37 ºC. Figures A and B show release properties of MTX and HCPT, respectively.


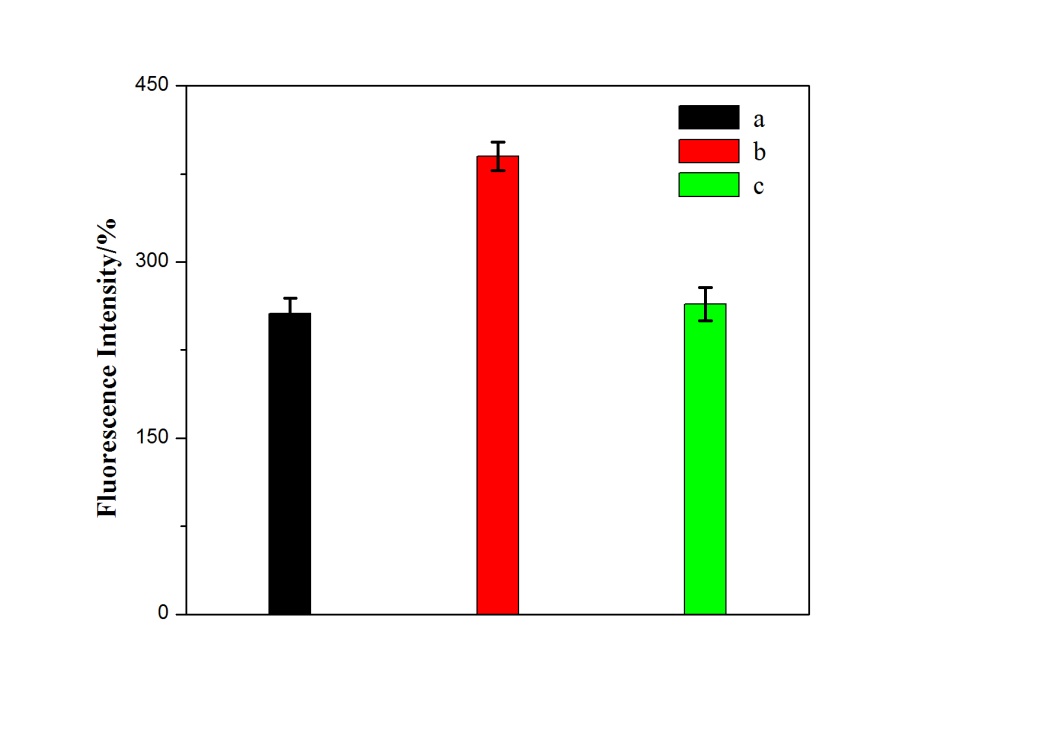


**Figure S7.** Fluorescence measurements of the HeLa cells incubated with HCPT-chisotan nanoneedles (a), dual-drug nanoneedles (b), and dual-drug nanoneedles in the presence of FA (c) over a 8 h incubation period at 37 °C, where P < 0.05.


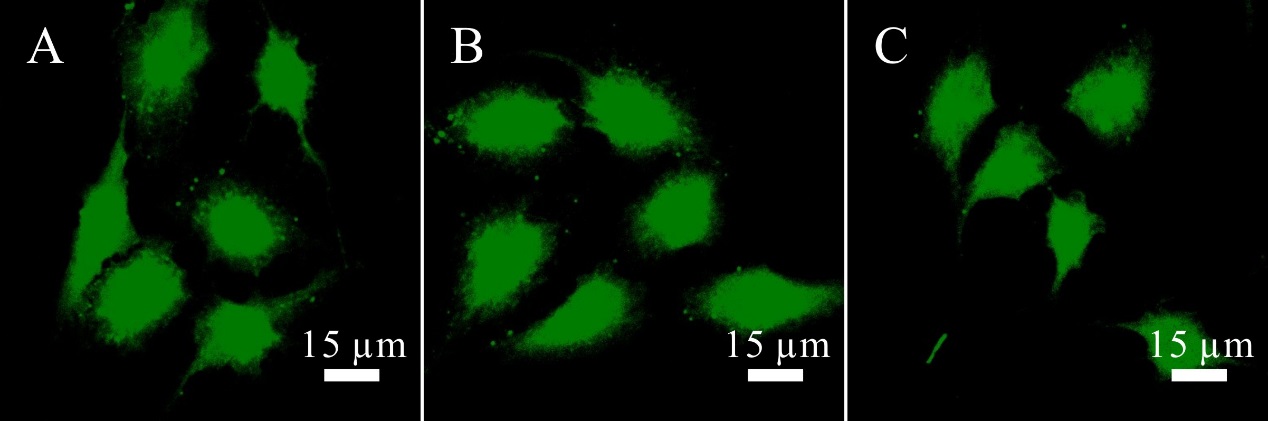


**Figure S8**. CLSM images of MG-63 cells showing intracellular drug delivery properties. Cells were incubated with dual-drug nanoneedles (A), HCPT-chisotan nanoneedles (B), and dual-drug nanoneedles in the presence of FA (C) over an 8 h incubation period at 37 °C.


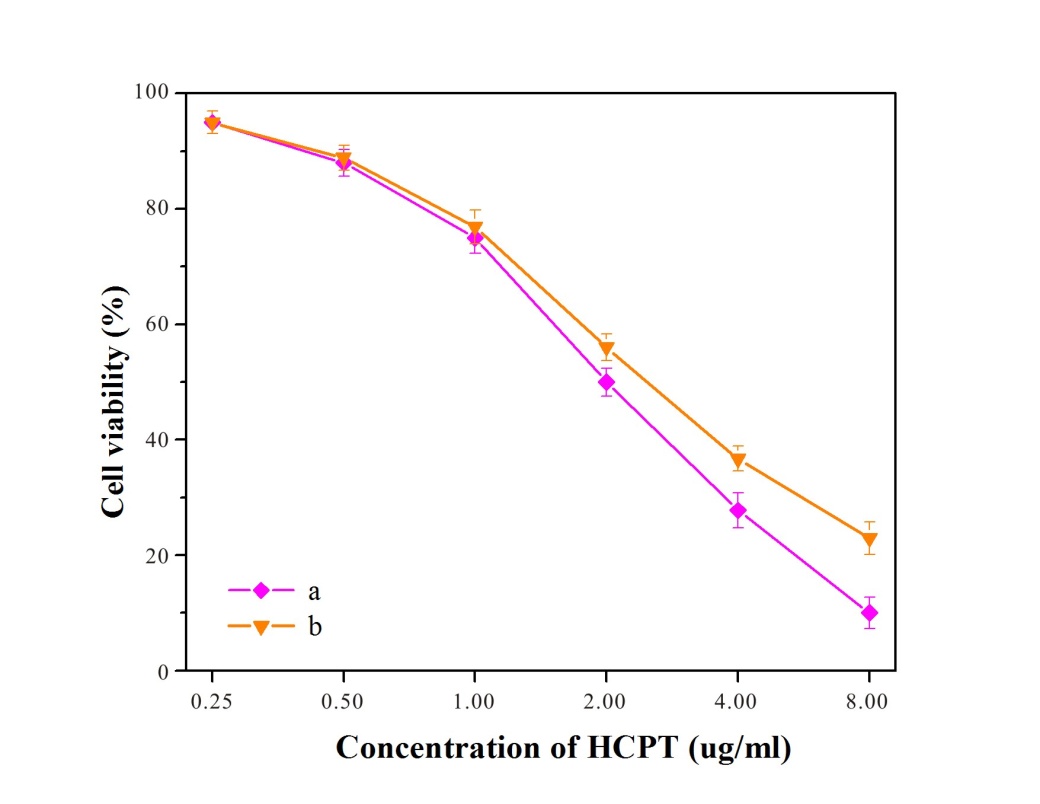


**Figure S9.** *In vitro* cell viability of the HeLa cells treated with dual-drug nanoneedles (a) and dual-drug nanoneedles in the presence of FA (b) after incubation of 24 h. P < 0.05.
